# Evaluation of a blended learning approach on stratified care for physiotherapy bachelor students

**DOI:** 10.1186/s12909-023-04517-5

**Published:** 2023-07-31

**Authors:** Mishael Adje, Jost Steinhäuser, Marjan Laekeman, Slavko Rogan, Sven Karstens

**Affiliations:** 1 Therapeutic Sciences, Department of Computer Science, Trier University of Applied Sciences, Trier, Germany; 2 Institute of Family Medicine, University of Luebeck, Luebeck, Germany; 3 Department of Physiological Psychology, University of Bamberg, Bochum, Germany; 4 Division of Physiotherapy, School of Health Professions, Bern University of Applied Sciences, Bern, Switzerland

**Keywords:** Blended Learning, Mixed-methods research, Stratified Care, Bachelor Physiotherapists, Low Back Pain, Kinesiophobia

## Abstract

**Background:**

Stratified models of care are valuable for addressing psychosocial factors which influence the outcome of patients with musculoskeletal disorders. Introducing such models in undergraduate training has the potential to propagate this knowledge with evidence and foster its implementation. The objective of this paper is to explore the perception and changes in the fear-avoidance beliefs of physiotherapy students participating in a developed blended learning course on stratified care.

**Methodology:**

A mixed-methods with a convenient sample of two consecutive cohorts were given a blended learning course on stratified care for patients with low back pain. The blended learning course comprised scientific rudiments and application of stratified care in clinical practice conceptualised using the KERN’ 6-step approach. The exam scores, perceptions, performance on self-reflection-tests and pre- and post-scores on The Tampa Scale for Kinesiophobia for Physiotherapists’ (TSK-PT) were obtained. After gaining clinical experience, participants were invited to discuss their clinical experiences and perceptions in workshops. The quantitative data was analysed explorative-descriptively. The qualitative data was analysed following an inductive coding system with constant comparisons.

**Results:**

Ninety-one participants consented to the evaluation (mean age = 22.9 ± 1.6 years), 66% were female. Exam scores correlated with time spent in training (r = 0.30) and scores on self-reflection-tests 1 and 2 (r = 0.40 and r = 0.41). Participants in both cohorts described the learning resources as promoting their interest in the subject (72% and 94%), up-to-date (91% and 93%) and helpful (91% and 97%). The fear-avoidance scores for participants decreased from 53.5 (± 9.96) to 40.1 (± 12.4) with a large effect size (d = 1.18). The regression model [F (2, 49) = 1151.2, p < 0.001] suggests that pre-TSK-PT and the interest of participants in the training predicted post-TSK-PT. The workshop participants (n = 62) all worked in clinical practice. Emerging from the analysis were 4 categories (evolving to maturity in practice, perceiving determinants of stratified care, strategising for implementation and adopting an outlook for future practice).

**Conclusion:**

The quality of engagement in learning, training strategy and interest in the subject contributes immensely to learning outcomes. This blended learning course was successful in reducing kinesiophobia and influencing the participants’ attitude towards care with the potential of being translated into long-term practice.

**Supplementary Information:**

The online version contains supplementary material available at 10.1186/s12909-023-04517-5.

## Introduction

### Background

The concept of ‘chronification’ in low back pain (LBP) connotes the transition from an acute to a chronic phase of this condition. It has been known to be catalysed by psychological processes [1, 2]. An example of this is “fear avoidance behaviour” which influences the development of the health status of patients with musculoskeletal complaints by discouraging activity, influencing treatment choices, contributing to the patients’ pain experience and fostering regression of the patient’s condition [3]. Studies show that these psychosocial factors when present in clinicians can inadvertently be reflected in their beliefs, attitudes, choice of treatment to administer and health care advice and ultimately have an untoward effect on patients’ conditions [4, 5]. Research shows that many acute LBP cases are self-limiting and problems resolved within the first 3 months however, about 10% of these cases become complex and progress to a persistent problem that leaves them physically impaired for a considerable duration [6].

It can thus be deduced from research that patients who posses similar characteristics such as their prognostic profile and are likely to respond to specific treatment can be categorised and targeted for treatment [7, 8]. A stratified model of care classifies patients based on the presence of inherent risk subcategories and targets treatment specific to such subgroups [9, 10]. There is evidence that standardized risk-specific stratified treatment (‘stratified care’) approaches could be superior to traditional physiotherapy practice for patients with LBP [9, 10]. Up-to-date therapy concepts developed and evaluated in the last 10 to 15 years build on these findings, hence psychological interventions have been designed to prevent chronicity and have proven effective when applied appropriately [5]. Such interventions have been incorporated into models that structure prognostic factors by the flagging system and used in physical therapy to target treatment processes [11]. One of such intervention is the Subgroups for Targeted Treatment (STarT-Back) approach. It categorises patients with non-specific LBP into low, medium, and high-risk by means of a guideline-recommended prognostic tool called the STarT-Back Tool (SBT) [12–14]. It further aims to deliver best-suited care integrating physical and psychological treatment approaches [15–18]

Although the biological model of health has traditionally been the focus of physiotherapy education, it is now commonly acknowledged that biopsychosocial factors have a considerable impact on musculoskeletal prognosis [19].

The barriers of cultural, personality differences and lack of motivation have been reported in recent times to negatively affect students learning. Traditional face-to-face teaching is regarded by some students as unstimulating since smartphones and computers have diminished their attention span. Amidst these challenges, physiotherapy educators face the constant challenge that students feel some concepts are not directly related to their carer requirements hence they pay little attention [20, 21]. To make learning more interactive and motivating, blending the traditional face-to-face instructional methods with an online component seems to provide some benefits. In combination with comprehensive early training of physiotherapists using theoretical concepts, blended learning and in-depth evaluation can have good potential to incorporate psychosocial management concepts into long-term clinical practice. This is possible since these young physiotherapists-in-training eventually evolve into full-fledged clinicians and contribute to shaping the future of physiotherapy practice [20–22].

Today, only a small percentage of physiotherapists in practice have the necessary training to incorporate psychosocial management concepts [23]. Additionally, there are lapses in undergraduate education in the aspects of scope of the training curriculum and practice components containing these concepts have not been fully integrated into the undergraduate education [23–25]. In a recent study, physiotherapists highlighted a gap in knowledge stating they would be unsure about physiotherapists competencies and consider training necessary especially for high risk patients [25]. To address these lapses, it is important that effective physiotherapy training determines the attention, receptivity, and satisfaction in relation to the social, cognitive, and emotional processes of both the individual and the group in the context of their learning environment [26]. The Community of Inquiry (COI) model of inquiry-based teaching and learning by Garrison et al., can thus form the foundation for shaping comprehensive physiotherapy training [26, 27]. This model focuses on the constructive views of experiential learning and describes the necessary components such as how deep and meaningful learning should occur. The focus is here on the education experience as occurring at the convergence of cognitive, teaching and social levels. Inquiry-based teaching and learning represents an important component in the learning process as well as an object of learning. Its origins took into consideration the use of individual experiences and the construction of individual knowledge structures as the key to engagement and learning success [28, 29]. This approach enables learning through cognitive engagement and helps to develop competencies in higher-order thinking [26]. Inquiry-based teaching focuses on providing meaningful opportunities to engage with the topic rather than giving direct instruction on the content as seen in passive learning. Based on these principles, a blended learning strategy based on the COI model of inquiry-based teaching was thus created for pre-graduate physiotherapy students to remedy the gap in knowledge.

### Objectives

The objectives were to explore the engagement and perception of pre-graduate physiotherapy students in a stratified care blended learning programme oriented on the COI model and characterise the changes in their fear-avoidance beliefs.

### Research questions

1. Was there a correlation between exam scores, self-reflection test scores and training level?
2. What effect does the training have on kinesiphobia in participants?
3. What variables influence the development of kinesiophobia?
4. What is the perception of students on the stratified care training?

## Methods

A mixed-methods approach was adopted, comprising pre- and post-quantitative and qualitative aspects over two semesters. The convergent parallel type of mixed method approach was chosen ‘to obtain different but complementary data’ on the topic of blended learning course on stratified care for physiotherapy participants [30]. This study required crucial information on the level of training participation, the effect on fear avoidance beliefs as well as the depth of understanding, reproducing and utilising learning in practice. Hence the mixed-methods approach was selected since it gives details more comprehensively than by using either quantitative or qualitative methods alone [31]. All students from a bachelors programme in physiotherapy from classes 2020 and 2021 were invited to participate. Students from other degree courses within the institution or from other universities were excluded from participation.

Participants were affiliated with different medical institutions around the region where they received training for their Diploma in Physiotherapy and were concurrently registered in the Physiotherapy Bachelor programme at the Trier University of Applied Sciences, Germany. They were invited to decide separately on participation in the quantitative and qualitative aspects of evaluation and could choose to participate in both. Participants consent was obtained individually in writing, there were no incentives provided and their confidentiality was ensured as all records were pseudonymously analysed.

In parallel to the first semester most of the participants took their state licensure exams, received their diplomas and began clinical practice while still in the bachelor’s degree programme. Quantitatively, the development of the students during the first semester of the programme was documented. These include training statistics, time spent engaging with training resources, number of attempts and scores on tasks. To increase the credibility of the results, workshops were conducted during the second semester. This was ideal for describing experiences with clinical implementation [32].

### Training development and design

This training on stratified care was developed with contributions from previous research, training, consultations and practice experiences gathered by the research team members [23, 24, 33]. It was conceptualised using the KERN’s 6-step approach [34] and based on the idea that entry-level physiotherapists must be equipped and trained to address patients’ musculoskeletal complaints in a structured manner as demonstrated by Ballengee et al., (2020) [22].

The content was prepared and hosted on an online platform ‘Open Online Learning and Training’ (OpenOLAT) and included webinars, self-learning sessions assisted by online materials and tasks, videos, quizzes and web-based mentoring. The purpose of this training was to equip participants with knowledge on the development and prognosis of non-specific LBP, the role of prognostic factor screening to identify and resolve these factors (Additional file 1). Further, participants were taught the impetus, scientific rudiments of stratified care and its application in clinical practice. Workshops serving as research and teaching components simultaneously complemented this by providing participants with the opportunity to critically discuss clinical experiences and perceptions. Participants could then reflect on their learning progress in relation to their clinical practice experiences and critically discuss biopsychosocial risk factors as considerations for the subsequent management of musculoskeletal conditions [22, 24].

### Training procedure

The training was in three consecutive sessions, with each session preceded and followed by a summary and preparatory task. The first phase focused on the fundamentals of stratified care. The basics of which were laid out in the 4th semester (summer semester) and enveloped in the larger credit-bearing module on assessment in clinical practice. The application in practice was promoted in a second phase in the 5th semester (winter semester) within the context of non-credit-bearing seminars. The contents of the theoretical phase were implemented exclusively digitally in two sessions. After a phase of three months giving participants the opportunity to gather experiences in clinical practice, the workshops were carried out. Moderated by a researcher, participants discussed key aspects of their perceptions and experiences, highlighting barriers, enablers and suggest strategies to purposefully reduce factors that inhibit implementation and make use of factors that promote it [34].

**Fig. 1:**
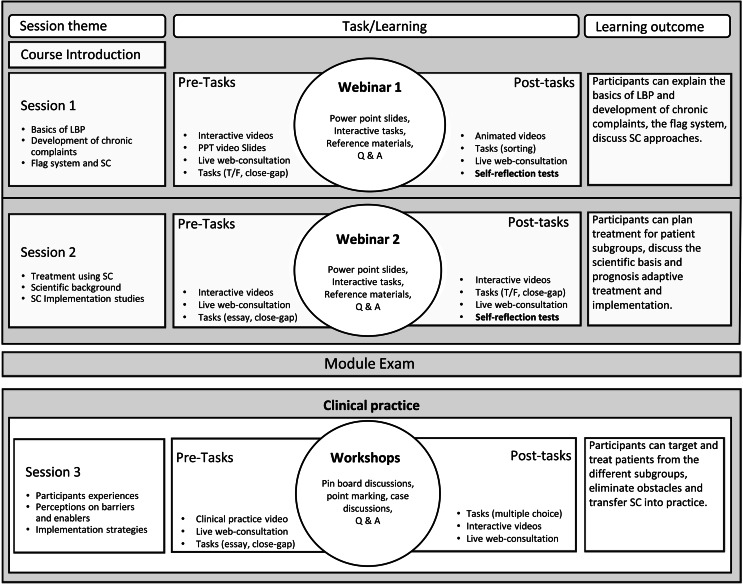
Training Structure LEGEND: SC; stratified care, LBP; low back pain, T/F; true or false questions

Figure 1 shows an overview of the programme structure provided to participants before the training commenced. At the course introduction, participants were instructed face-to-face on getting started with blended learning, the aims of the training and the evaluation. Each session comprised preparatory tasks and post-tasks that adopted custom-designed interactive videos, pictures and text resources, Intermittent tests with essays, multiple choice, sorting-type questions with case studies, and additional reference materials.

Webinars were organised, and sandwiched between the pre- and post-tasks to assure learning progress. Additional help was provided via online live consultation sessions offered by the course moderator weekly for discussions. Answers to participants’ questions from these sessions provided by the moderator or fellow participants were compiled and available online as ‘Frequently Asked Questions’. Self-reflection tests were provided at the end of each of the first two blended learning sessions. These were considered specific milestones of the study and didactically important components aimed at self-checking participants learning progress. During these self-reflection-tests, participants were required to complete a short quiz as a summary of each session thereby providing a basis for progression to the next training session. The quizzes were multi-component tests that reflect students understanding of the training and assist them to assess the training components. The high degree of self-directed learning allowed the students to flexibly design their learning process. The module exams took place after the first two sessions ushering participants into clinical practice while engaging in the third training session. The core of the third session was the workshops (which had an evaluative and at the same a didactic purpose). Here, pinboard discussions were used. Key ideas from the discussions were written on cards and placed on pinboards, and interactions with other ideas were created. Participants provided immediate feedback on pin-board items given by participants a via point system showing their opinion of acceptance or rejection. The training was homogenous for both cohorts with similar content and intensity. Participants in both cohorts thus completed a combined workload of 35 h spread over 6 months from 2020 to 2021 respectively.

### Quantitative description of the development of physiotherapy students participating in the blended learning programme on stratified care

#### Quantitative data collection

The Tampa Scale for Kinesiophobia for Physiotherapists (TSK-PT) was used to obtain participants’ levels of kinesiophobia pre- and post-training [35, 36]. Therapists can achieve a value between 17 and 102 points on the TSK-PT scale. A higher score represents a higher level of kinesiophobia. The use of this tool is due to the fact that kinesiophobic beliefs and attitudes of clinicians have been shown in studies to influence patients and need to be properly addressed in training to enhance competency of physiotherapists in biopsychosocial treatment [37–39].

Training statistics including scores on tasks, number of attempts, and time spent on each training material were obtained to determine the level of participation in training. During the training, involvement in the online activities was documented pseudonymously by counting the tasks completed and at the end of the term exam scores (written module examination). The training impressions were evaluated using predefined scales. Participants were asked to evaluate the following three main aspects of the training: (1) the form and structure (4 items), which covered the resources used and the course content; (2) the learning success (3 items), which evaluated how well the participants felt they learned and whether their learning aligned with the training objectives; and (3) the relevance (1 item), which examined how the participants felt the training was relevant and affected their interest in the topic. Each scale has five points from ‘strongly agree’ to ‘strongly disagree on a Likert scale [40].

#### Quantitative data analysis

Demographics such as participants sex and age along with further data such as number of tasks completed, scores, attempts and time spent on tasks were analysed descriptive-exploratively.

Point measures and measures of variability were calculated and complemented using figures. Repeated measures ANOVA analysis was carried out for TSK-PT outcomes pre- and post-training. The effect sizes were determined using Cohen’s d criteria [41], where the small effect was (d = 0.2), medium (d = 0.5), and large (d = 0.8 or higher). Correlation analyses were done using Pearson’s coefficient to determine the strength and direction of the association between training components and final assessment performance. Studies show that variability in training could lead to inconsistencies in learning outcomes [42]. The correlation coefficients were stipulated according to standard; 0.00 to < 0.20 is regarded as very weak positive correlation, 0.20 to < 0.40 connotes weak positive correlation, 0.40 to < 0.60, stands for moderate positive correlation, 0.60 to < 0.80 represents strong positive correlation and 0.80 to < 1.00 represents very strong positive correlation [43].

A multivariate linear regression analysis was carried out for selected variables. The variables ‘time spent with the learning resources’, ‘number of attempts on tasks’, ‘completed tasks’, ‘scores on tasks’, ‘scores on self-reflection tests’ and ‘perception of learning’ were selected. These were chosen based on studies which shows that student’s engagement with the learning materials, interactions, motivation time and effort are key factors to attain successful learning [20, 21]. There is also evidence that gender diversity is a major characteristic in higher education [20] hence the variable ‘sex’ was included. These variables were included if they reached a pre-set inclusion significance level (p ≤ 0.30)[44]. This was done using the backward elimination method with the dependent variable being the post-kinesiophobia scores of participants. The dependent variable post-kinesiophobia was considered a viable measure of participants outcomes after training since it reflects an overall change in participants’ status. All analyses were performed using SPSS IBM V26. The level of significance was set at p < 0.05. The STROBE checklist was used for reporting the quantitative aspect.

### Qualitative determination of participants perceptions of the programme and implementation in clinical practice

#### Qualitative data collection

Eleven [11] qualitative workshops were conducted serving a dual purpose of teaching and research as described previously in Additional file 1. They took place three months after the first and second training sessions. To progressively guide the workshops and for data collection, a semi-structured interview guideline was developed using a standardised criterion procedure by Brosziewski and Helfferich [45]. It was advised that participants bring some theoretical understanding of the issue to the interviews, have a pleasant introduction in person, and gradually become accustomed to the interviews using semi-structured questions. Three key questions were developed with adaptations from studies by Krueger and Homberg et al. [46, 47] with further maintenance questions and follow-up questions (Additional file 2: Interview guideline). In line with the iterative process of qualitative research, the guideline was modified when new research areas were identified during the process (Additional file 3: Qualitative findings from Workshops).

The interview questions covered the subject of stratified care, the interviewees’ opinions of the stratified care training, and the information and skills they acquired from the training. The questions were ordered in a way that would encourage participants to consider how their knowledge might be put to use [48].

Data was collected during the workshops employing hand-written dictation capturing participants’ direct quotes done independently by two researchers (MA and SK), pinboard cards, and posters [49, 50] in a relaxed, comfortable classroom setting and the absence of any non-participants. MA is a physiotherapy PhD student with experience in musculoskeletal health; SK, is a professor of physiotherapy with experience in mixed methods and health services research. Both researchers have certified competency in stratified model of care.

#### Qualitative data analysis

The gathered data was put together including feedback from participants and preparatory self-reflection exercises. The derived data were read through several times by two researchers, and code labels were developed directly from the data. The coding tree was designed, checked with the Consolidated Framework for Implementation Research (CFIR) for completeness [51] and modified by two researchers who held several discussion sessions to reflect and compare coding. Relationships between the open codes were identified and common connections were established to create base-level categories. They were further combined into higher-level categories. Constant comparisons were used to develop connections, and relationships and discover new areas of inquiry until saturation. This was considered as the point where the new collection of data did not shed any further light on the issues under investigation [52].

For coding, an inductive category system was used following the method described by Corbin and Strauss aided by R-Qualitative Data Analysis (RQDA) software [53, 54]. This technique, an iterative process of qualitative research analysis, made sure that participants had a significant influence on the development of the research process and interview guidelines. Feedback about findings was given to each cohort in the form of group discussions for each set on the quantitative and qualitative aspects and further inputs were obtained.

The qualitative study report was provided following Standards for reporting qualitative research (SRQR) criteria. The entire mixed-method was described in a study protocol and registered on the Registry of Efficacy and Effectiveness Studies (Registry ID: 10,300) before commencement of this study.

## Results

### Describing the development of physiotherapy students participating in the blended learning programme on stratified care

Of the 91 eligible students from both cohorts, 62 consented to participate in the evaluation giving a response rate of 68%. However, 29 (32%) opted not to participate in the evaluation. The ninety-one participants in two cohorts consented to the training (n = 91), aged 21–26 (M = 22.9, SD = 1.6). Both cohorts had the same proportion of females (66%). The demographics of training participants are shown below (Table 1).

**Table 1:** Participants Characteristics LEGEND: Class2020; first cohort, Class2021; second cohort

Characteristics	Participants Quantitative			Participants Quanlitative		
	All	class2020	class2021	All	class2020	class2021
**Sex**						
Male n (%)	31 (34)	15 (34)	16 (34)	20 (32)	10 (36)	12 (35)
Female n (%)	60 (66)	29 (66)	31 (66)	42 (68)	18 (64)	22 (65)
Total n (%)	91 (100)	44 (100)	47 (100)	62 (100)	28 (100)	34 (100)
**Age (in years)**						
Mean (SD)	22.9 (1.6)	22.5 (1.2)	23.3 (1.8)	23 (1.9)	22.1(0.1)	24.8(1.9)

In describing participants engagement with training resources, training statistics are indicated for both cohorts. Participants time spent with resources, number of attempts, scores on tasks and self-reflection tests, kinesiophobia before and after training, exam scores and perception of the training yielded substantial results.

The mean scores and attempts per task were similar for both cohorts however, class 2020 spent slightly more time considering the training materials (M = 12710.5, SD8417.8) compared to class 2021 (M = 9721.2, SD7434.4).

Conversely, class 2020 had fewer attempts on tasks (M = 12.2, SD7.6) compared to class2021 (M = 13.9, SD7.6) and lower mean scores (M = 51.3 SD21.5) compared to class 2021 (M = 56.5, SD18.4).

Describing the final exam performance in both cohorts. Of the 91 participants in the training, 62 took part in the module exam. The maximum achievable score was 30 points (20 points for Part A and 10 points for Part B). The mean score shows that class 2020 scored slightly higher (M = 18 ± 6.1) than class 2021 (M = 16 ± 4.6). However, there was no statistically significant difference in final exam scores (p = 0.16).

There was no statistically significant difference in total time spent on tasks (p = 0.17), scores of training tasks (p = 0.06), attempts between both groups (p = 0.16). There was also no statistically significant difference in the time, scores and attempts expended in the self-reflection tests (p = 0.05, p = 0.31, p = 0.59) (Additional file 4: T-test for significant difference in training statistics between groups).

### Kinesiophobia

In describing changes in participants’ kinesiophobia after training a pre-post evaluation shows the level of fear-avoidance measured using the Tampa Scale for Kinesiophobia. This shows a statistically significant difference in scores obtained post-training as shown below in Table 2.

Before the training, the mean total value for both cohorts was 53.5 ± SD 9.96, after the training, the value was reduced to 40.07 ± SD 12.45. Class 2020 had a reduction of 14.97 while class2021 had a reduction of 11.84. The effect size was large with Cohen’s d value at 1.18 (Table 2). There was a difference between pre- and post kinesiophobia scores of both cohorts but there was no statistically significant difference between kinesiophobia scores of class2020 and class2021.

**Table 2:** Difference in Kinesiophobia Between Groups LEGEND: Cohen’s d = effect size: small (ds = 0.2), medium (ds = 0.5), large (ds = 0.8 or larger) * alpha level p < 0.05, Class2020; first cohort, Class2021; second cohort

Kinesiophobia	Groups	Mean (SD)	Total Mean (SD)	Sig.	Cohen’s D	95% Confidence Interval	
						Lower	Upper
PreTest	class2020	55.53(9.95)	53.47(9.9)	< 0.01*	1.18	51.130	55.816
	class2021	51.42(9.69)					
PostTest	class2020	40.56(12.79)	40.07(12.5)			37.082	43.060
	class2021	39.58(12.28)					

**Fig. 2:**
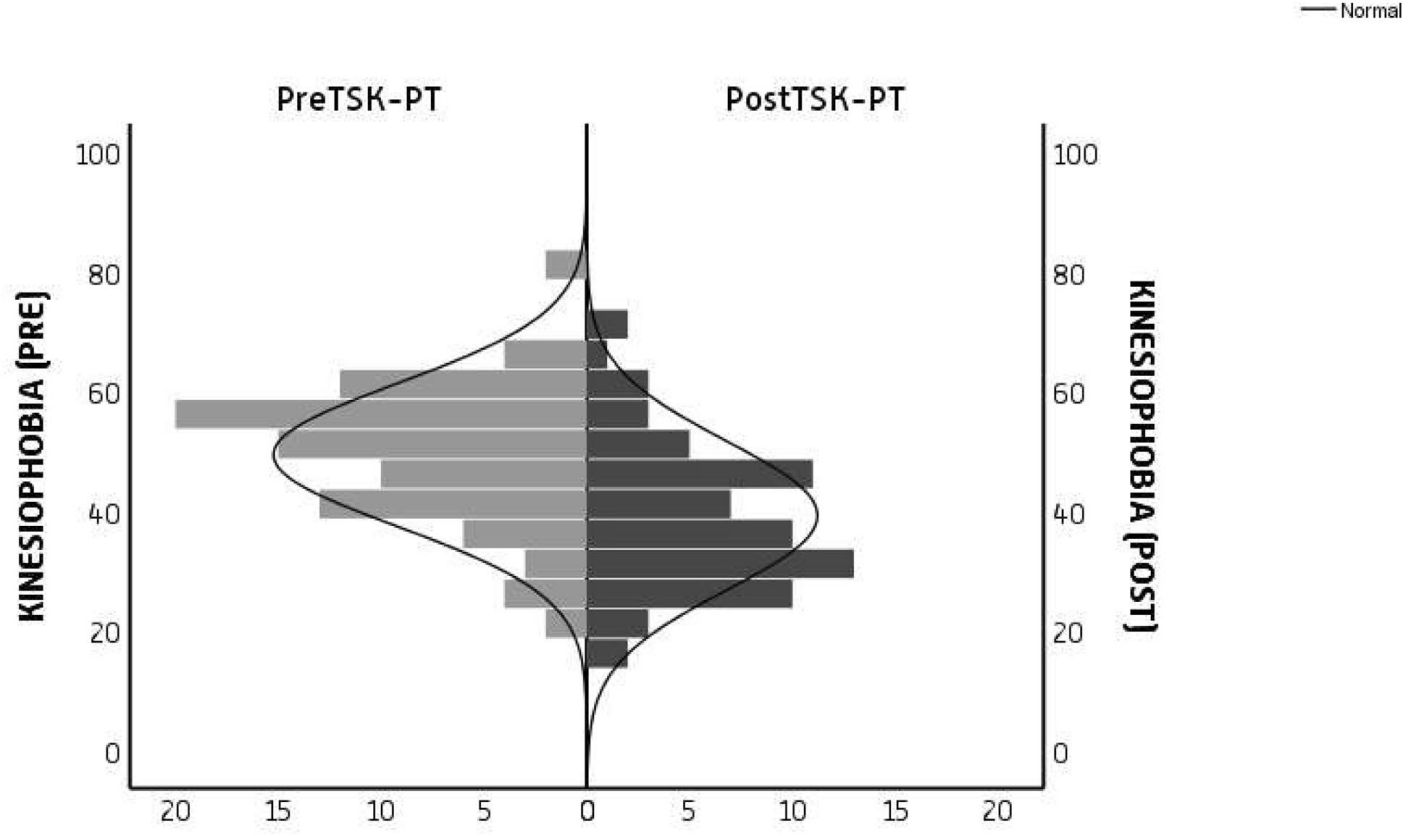
Change in Participants’ Kinesiophobia Scores Measured by Tampa Scale of Kinesiophobia for Physiotherapists before (PRE) and after (POST) training LEGEND: PreTSK-PT; Completed by participants before intervention, PostTSK-PT; Completed by participants after intervention

The histograms for TSK-PT show pre-TSK-PT scores peak towards 50(49%) and post-TSK-PT peaks around 40(39%) (Fig. 2).

### Perception

The perception of training presents graphical similarities as shown in Additional file 5. In describing the relationships between training and exam performance in both cohorts Pearson’s correlation coefficient was employed. The result shows there was a moderate to low correlation between training and exam scores with a significance level of p < 0.05 (Table 3).

There was a moderately positive correlation between scores on self-reflection tests (SRT1 and SRT2) and exam scores in both groups (r = 0.40, r = 0.41) (Table 3). The time spent on tasks correlated low with exam scores (r = 0.30) as depicted in Table 3. Perception of training had a low negative correlation to exam score (r=-0.35).

**Table 3:** Correlation of Exam Score with Key Training Tasks and Perception LEGEND: *Correlation is significant at the 0.05 level (2-tailed). Perception(mean) of all eight perception items from participants in both sets

Exam Score		Total (n = 62)			
		Pearson Correlation	Sig.(2-tailed)	95% Confidence Interval	
				Lower	Upper
Score	SRT1	0.41*	< 0.01	0.18	0.63
	SRT2	0.40*	< 0.01	0.20	0.59
Time spent		0.30*	0.02	0.03	0.58
Attempts		0.24	0.08	-0.08	0.44
Perception (mean)		-0.35*	0.01	-0.57	-0.09

### Predictors of participants kinesiophobia outcome after training

All pre-identified variables of interest were tested on a univariate level using univariate linear regression. All variables with potential influence on the dependent variable (post-TSK-PT), the outcome of participants kinesiophobia. Seven variables in Table 4 reached the set cut-off significance level (p ≤ 0.30).

A regression model was therefore selected using predictor variables reaching the pre-set cut-off point. To achieve this a multivariate linear regression was carried out using backward elimination. The dependent variable post-TSK-PT was regressed on the predicting variables reaching the cut-off point. Of the seven variables, Pre-TSK-PT and EV08 (The course promoted my interest in the subject) significantly predicted post-TSK-PT F (2, 49) = 1151.2, p < 0.001. The influence of these variables on post-TSK-PT is shown for pre-TSK-PT as (b = 0.67, p < 0.001) and for EV08 as (b = 2.89, p = 0.02). Moreover, the adjusted R^2^ Square for this model R^2^ = 0.27 depicts that the model explains 27% of the variance of post-TSK-PT.

**Table 4:** Variables in the Regression Model (Univariate) LEGEND: Dependent variable; Post-TSK-PT Score, *Sig of model, Scores; Scores on tasks incl. SRT, Time; Time for all tasks incl. SRT, SRT; Self-reflection test, EV; Evaluation question, ^+^ Variables reaching set cut-off significance level

Variables	Sig
1. Pre-TSK-PT^+^	< 0.01*
2. Time spent on tasks^+^	0.17
3. Number of tasks completed^+^	0.21
4. Sex	0.38
5. Attempts	0.86
6. Total SRT Attempts	0.75
7. Total SRT Time	0.98
8. Groups	0.75
9. EV01: The material was appropriately illustrated^+^	0.20
10. EV05: The relevance of the teaching content offered was high^+^	0.09
11. EV07: I rate my learning gain from this course highly^+^	0.30
12. EV08: The course promoted my interest in the subject^+^	0.10

### Participants experiences with implementation in clinical practice and perceptions of the programme

A total of 62 students participated in eleven workshops. Participants were divided into; five (class 2020) and six groups (class 2021) each made up of 5 to 7 participants per workshop. Each group engaged in a single workshop lasting an average of 2 h.

Participants were 20 males and 42 females, with a mean age of 23 (± 1.9) years. Before participation in the workshop and after receiving their clinical licence, all students spent time in clinical practice. The workshops generated 70 pages of transcripts, 18 board posters and 658 board cards. The perspectives of participants on the implementation of the training content were captured in four main categories and eight sub-categories shown in Table 5. Codes that exist within these sub-categories have quotes that describe them (Additional file 6: Inductively developed strategies for implementation from Workshops and Additional file 7: Inductively developed categories, sub-categories, codes and quotes from Workshops).

### Evolving to maturity in practice

This category deals with participants’ personal experiences in relation to the training content, challenges faced during clinical practice, personal and environmental issues and how they are developing to maturity in their practice.

**Table 5:** Inductively Developed Categories and Corresponding Sub-Categories LEGEND: SBT; STarT-Back Tool

Categories	Sub-categories
Evolving to maturity in practice	Individual demands Self-advancement
Perceiving determinants of stratified care	Factors affecting use of the SBT Factors affecting use of the approach
Strategising for implementation	Adaptive strategies Targeting stakeholders
Adopting an outlook for future practice	Impression on training Resolve for application

#### Individual demands

Some participants explained that since they are relatively new in practice not many opportunities are available to use the approach. Everything seems new and they need some time to slowly get used to it. Additionally, the work stress, time constraints and situation with COVID-19 restricted their efforts to utilise the approach. Some explained for instance that group therapy sessions are not possible, and contact limitations further restrict the possibilities and limit patient turnout.

*G3P2: I could not use the approach due to work stress. I can’t spend much time in treatment.

G8P4: The corona situation makes it more difficult in the clinics.

*G-Group, P-Participant, SB-STarT-Back

#### Self-advancement

Some participants said they individually found ways to improve their practice in simple units. They have seen improvements in parameters related to the training as aspects of communication, attentiveness to yellow flags and the focus on increased activity. For instance, with improved communication during practice, they described noting when the patients gave comments that reflected yellow flags like hypervigilance and catastrophising and have learned to mentally categorise patients to educate them.

G5P3: my method of communication changed

G2P4: I have had more experiences in the past few weeks. After the training, I have learned to listen differently.

### Perceiving the determinants of stratified care

Here, participants shared their perceptions and ideas about the training content. From a third-person point of view, they gave their thoughts on the positive and negative determinants of the SBT, its approach and its implications for stakeholders.

#### Factors affecting the use of the SBT

For the tool, a key aspect seen by some participants is its simplicity, they related that the SBT is simple and well organised. Some felt it was additional work or inconvenience compounded by the traditional disuse of tools in clinical settings and having to balance patient load with questionnaires and sub-grouping.

G2P3: good and well-organised tool, it is well ordered and arranged

G1P4: on the other hand, it is additional work for clinicians and physiotherapists

#### Factors affecting the use of the approach

Participants here spoke about their ideas about the STarT-Back (SB) approach. Although they had a positive perception of the approach that it **saves time**, there were quite a number of challenges. Some complained about **time** for the use of the approach, and expectations from patients.

G3P2: positive aspects of the approach are that it saves time and is efficient

G2P4: Time constraints, longer treatment time (as seen in the SB approach for high-risk patients) usually not available.

As with the aspect of time, participants related that some other factors have dual effects, acting as facilitators to enhance the implementation of the SB approach as well as barriers to implementation. Participants considered that physicians and colleagues who are enlightened and familiar with the approach and evidence-based practice (EBP) could act to enhance its use. Concurrently, participants opined that the patients’ attitudes when educated could facilitate the implementation of the training content while those with unhelpful beliefs foster resistive attitudes. Participants further opined that physicians and colleagues who are unfamiliar with the approach or EBP in general, or who are changing attending therapists might act as barriers to its implementation putting forth some resistance in their place of practice. They reasoned that the process of frequently alternating therapists perhaps with differing ideologies might affect treatment objectives, procedure and hence implementation.

G10P2: characteristics of the doctor-is he knowledgeable or not. Knowledge education and exposure helps

*G3P4: Support from colleagues are enablers. I find the relationship with EBP interesting and I’m sure other colleagues will appreciate another perspective*.

*G4P5: Patient compliance. Some patients could be resistant to change in treatment protocol and ideas.*

*G3P5: In the clinics therapists change [new therapist for a patient already in treatment], can influence outcome and use.*

There was also the issue of income mentioned by participants. On the one hand, some saw the approach as cost-effective for patients, since patients in the low-risk category receive one session of treatment followed by self-care, they save on treatment and commuting costs. Others saw it as reducing income for the therapists since the low risk patients have fewer treatment sessions but considered that its positive outcome might compensate for this.

G3P5: Positive aspect was that the approach is cost-effective.

G3P3: fewer patients means less income

G10P4: Monetary loss gains with more chance of success with treatment

Participants spoke about frustration with the current practice condition and a need for change. In their opinion, it was a factor that could be a stimulus for change, hence the possibility to overcome this by introducing specialised training on psychosocial care and education. These key aspects they believe could positively affect implementation.

G5P6: Physiotherapist point of view, criticism of the existing system, frustration with some therapy treatment approaches.

G5P2: Currently physiotherapy isn’t doing as well as it should.

G4P2: Physiotherapists who do this training do better with the tool and practice of this approach.

### Strategising for implementation

In this category, participants shared their ideas on strategies to combat challenges identified as hindrances to implementing training content. They discussed approaches to utilise enablers with the aim of applying ideologies learned from the training in clinical practice (Additional file 4: Inductively developed strategies for implementation from Workshops).

#### Adaptive strategies

Participants here suggested different strategies that involve using the questionnaire, like *Issuing SBT before the first treatment or electronic automation of the process.* Since patients are often in the waiting room this suggestion implies they fill the questionnaire there or at home. Therapists could *use familiar aspects*, cherry-picking areas in the questionnaire for use in anamnesis. Participants think this could work well if selected aspects of the approach could be integrated, with proper organisation of routines *in the clinic.* Participants had the impression that doing this would require sufficient co-operation from therapists and patients. Hence, they suggested using the documented results supporting this approach, *standardised assessment materials* would resolve the challenge of changing physiotherapists. Further, *input from p*atients and colleagues would help to convince them and overcome their hesitation.

G2P4: fill out the questionnaire on or before first contact

G6P2: Integration of questionnaires in clinical routine is an enabler.

G4P3: Documenting outcome after using it in clinical practice.

#### Targeting stakeholders

In their view, interprofessional collaboration, educating patients by explaining to them and compromising on certain aspects of care if the patients resist can be the best way to reach a consensus. Participants in the suggested training on the use of the tool should be done for clinical physiotherapists as part of some form of continuous professional development or quality improvement programme. This in their opinion is a strategy that ensures capacity and skill to the users.

G4P1: A lot of explaining as understandable as possible for the patient.

G2P4: compromising approach to the activity if the candidate refuses at first

G2P5: Uniform approach of the therapists by internal training

### Adopting an outlook for future practice

This category deals with participants’ impressions of the training content they received. They gave their opinions on the strengths and weaknesses of the training, their perceived potential for influencing their clinical practices and the outlook they plan to adopt in their future practice.

#### Impression on training

Participants felt strongly that the training was interesting and beneficial, especially the self-reflection aspects. They related that it had rich content, and has the potential to boost their confidence in practice and help with organising themselves while getting into practice as new clinicians. The workshop aspect was seen as further consolidating the knowledge gained in class. However, some aspects were pointed out that needed adjustments. Participants opined that the training should be broadened and integrated into other aspects.

G5P4: directs young physiotherapists. It boosts the confidence of the therapist

G5P2: positive aspects are that we worked on a lot of topics by ourselves

G6P1: Need a bigger forum to discuss this involving other specialities and stakeholders.

#### Resolve for application

Participants appreciated the fact that the training content helps communication, treatment planning and fast-tracks decision making. They resolved to apply these aspects in practice to improve their communication with patients and prepare themselves for practice through self-reflection.

G5P1: Communication from patients, contextual relevance, and communication with other patients was an interesting aspect.

G2P1: A positive factor is planning therapy, being prepared to use the tool and hand it to the patient gives you a better idea of how to plan patient treatment.

## Discussion

This study aimed to explore the participation and perception of pre-graduate physiotherapy students in the stratified care blended learning programme oriented on the COI model and characterize the changes in their fear-avoidance beliefs. It revealed how the students developed with the programme and provided insight into the quality of training, influence on participants’ beliefs and potential in clinical practice.

After successful participation in the training, students reflected on their practical actions and the therapy modules developed for stratified care. They reported that it led to a better understanding of stratified care and helped them notice and handle their fear-avoidance beliefs. This is in tandem with the ‘theory of experiential learning’ by Kolb and Kolb’s, where concrete experience and the opportunity for reflective observation are provided to optimise learning and foster the transition from learning to practice [55, 56].

The **quantitative aspect** of this project reveals that the learning materials and methods employed in this study did not differ in quality between cohorts. The statistics show that participation in the aspects of scores on tasks and exam scores had no significant difference between cohorts. Findings also reveal that when the self-reflection tests were considered in terms of participation and scores, the cohort 2020 did not differ statistically from the cohort 2021. It can thus be deduced that participation and outcome levels were maintained. This suggests that when the quality of training is maintained and monitored similar performance can be expected [57]. Findings from this study also show a significant correlation between task scores and final assessment performance. Time spent in training correlated significantly with the final assessment. A possible explanation could be that the training was well assimilated and with more consistent time and effort participants might have developed better competency. This finding thus suggests, that aside from scores, time spent on training materials should be considered when aiming for successful learning as seen in literature [58].

As further seen in the quantitative aspect of this study, participants’ fear of movement (kinesiophobia) decreased significantly post-training compared to pre-training. It could be interpreted that participants beliefs relation to their patients fear of movement and physical activity improved. Hence this could be reflected in their advice to patients and choice of treatment modalities. This decrease could be explained or influenced by different possibilities; the benefits gained from the training, their perception of the training further corroborate this postulation [59]. Participants described the training on stratified care as meaningful, interesting and motivating. They agreed that this training should be continued as it contributes to optimising professionalism. Research in agreement with this shows that experience and knowledge of the profession are key factors influencing professional identity development at early training phases in the health sciences [60].

In the **qualitative aspect** of this study, participants described their experiences and how their practice evolved with the training on stratified care they received. Some participants used aspects of stratified care modified in individualised ways in their practice with varying results. Although some complained of too few patients and the lack of opportunity to showcase learning, a majority reported that their perspective changed as well as aspects they paid attention to during practice. Their awareness of psychosocial factors such as fear avoidance and their ability to handle it with communication and self-reflection were areas that notably improved in their perspective. Literature suggests that students are more aware of learned practical components when they experience them and are better prepared to handle them [55, 56]. The highest consensus in this study was participants’ resolve to pay attention to their fear-avoidance beliefs in communication as it plays a major role in treatment outcome. This was a key aspect of learning and has been put into practice by participants to a considerable extent as their experiences show. These findings corroborate what research shows, a study by Maxwell et al., (2009) reveals that physiotherapy students considered communication and observation important skills and vital learning areas. However, despite the theoretical base in psychology, they had developed few communication skills and had limited knowledge of its implications for practice [61]. This points to the ongoing discussions on the knowledge and skill gap in the undergraduate training programmes in Germany seen in the literature [23]. Since about 95% of physiotherapy students are trained at a vocational level, many do not receive intensive training and mentorship on the use of prognostic tools and ‘methods of communication to address unhelpful beliefs and illness behaviors’ needed to handle patients with psychosocial risk factors [23, 62].

Integrating the qualitative and quantitative results reveal complementary findings. Participants’ interest was evident from the levels of participation shown in the training statistics in both cohorts. This is corroborated by the perception of above two-thirds of participants in both cohorts that the training ‘furthered their interest in the subject’. It was therefore deduced that interest in the subject predicted the outcome of the training. The self-reflection tests enabled participants to deeply think about the training content and relate them to real-life situations. It was revealed that these self-reflection tests correlated with training levels and exam scores, hence good participation here equipped them to master the content of the material, identify and discuss potential factors affecting training content in practice providing strategies for tackling them in relation to the aims of the training. They relate during the qualitative workshops that this self-reflection aspect was key in helping them translate theory to practice and boosting their confidence in preparation for practice [23].

Complementary findings were also seen in the aspect of kinesiophobia. Quantitatively they had a significant positive change in their fear-avoidance beliefs. Qualitatively, they showed a potential ability to identify, handle yellow flags and relate how communication could be dynamically integrated into clinical practice.

### Outcome of self-reflections

During the workshops, participants self-reflected on how the blended learning training content they received could relate to clinical practice. Insight on their perceptions in light of their clinical exposure was given in addition to strategies to put training content into practice. For the SBT, an electronic version of the tool might further simplify usage and influence the therapists’ choice of the assessment tool. Participants perceived inherent frustration with current practice patterns and suboptimal results. Research emphasises that this existing frustration creates a ‘need for change’ which is necessary for successful implementation [23, 51]. Hence, a positive treatment experience, outcome and feedback from patients will be potentially effective in convincing colleagues, seniors and other patients to adopt the approach. Interpersonal factors having to do with physicians and colleagues play a two-pronged role. Studies show that when stakeholders are educated and informed they support evidence-based approaches as shown in this study [63].

Participants reckoned that awareness and skills needed to apply the SBT are key to successful and effective use of the approach [64]. Since many physiotherapists in Germany do not have a bachelor’s degree, they opined that there should be mutual training to inform colleagues. In practice, the therapist would need to reflect on how to adapt the SB approach in routine practice and initiate a ‘buy-in’ for stakeholders [23].

Time and income were seen as a common denominator in numerous studies on the implementation of stratified care [23, 63]. Interestingly, these factors were perceived by participants as relevant even to physiotherapists-in-training as they evolve to maturity in practice. Though the SBT could help save some time, participants felt this could not be commensurate with the time and effort required for high-risk treatment and assessment. Although, participants perceived this approach might reduce income they still believed it was advantageous because patients are more likely to return and give recommendations for a care center when they receive effective treatment. Recent studies noted that patients require conviction, quality communication and time for successful implementation. Participants in this study suggested physiotherapists should not only educate and inform patients but also have their part in the decision-making, and planning process and reach a consensus with patients [24, 65]. This should involve compromise and conviction which might be challenging but vital to achieving successful implementation [66].

#### Implications on teaching and learning

The implications are numerous for the development of ongoing teaching and learning in practice-oriented modules for use in broader settings involving health professionals. It can be inferred from the findings of this study, that focusing on stimulating the interest of participants and varying the training resources, contributes to successful training. This was exemplified by the dynamic use of learning materials and blended learning format. Since this training was more self-learning and less classroom participation, students could schedule their learning periods at their convenience. Existing research corroborated by this study shows that the flipped method is a potentially effective teaching method and can produce significant learning results as high grades can potentially translate into practice competency [67].

For practice training of clinical physiotherapists in various settings and contexts the design and execution of the workshops were seen to be effective derived from literature which suggests a focus on equipping participants to adapt and adopt learning into practice [23, 68]. In addition, participants suggested that physiotherapists and trainers should also make an effort to identify and use facilitators within the practice content to bridge the knowledge-practice gap. In their opinion, as an example, they noticed the SBT and approach already have the advantage of being simple and easy to use, helping with treatment co-planning and enhancing the decision-making process. This was similarly noted even by experienced practitioners in a recent survey among physiotherapists working in Germany. They reveal the implication of the complexity of an assessment tool as one of the most prominent reasons for its limited use [64].

### Strengths and Limitations

In contrast to previous work on the implementation of stratified care in routine practice, the participants of this workshop had experience in clinical practice after participating in the training [23]. Nevertheless, they were new to clinical practice hence their time spent in the clinic and possibilities for using the approach varied greatly and could be seen as a limitation. This was seen in their experiences being narrow in range. However, studies show that fear-avoidance beliefs mostly develop from early practice stages, and undergraduate training is sustained through practice [35, 69, 70], hence a very relevant time point for intervention was met. Their experiences were interesting and revealed the challenges for young physiotherapists that are relevant for instructors. The results confirm that the workshops were done at a very relevant time in participants’ careers. They had just received their first clinical experience, hence providing the opportunity for the researchers to gain insight into their first impressions of clinical practice to guide them as they establish routines.

A few who participated in the training did not take part in the workshops. These were students who didn’t enrol in the module which encapsulated the workshops had already completed this aspect or planned to enrol at a later time point. All participants were however given the opportunity to provide feedback after the training and contribute.

The participants preferred not to have the discussions audiotaped during the discussions, hence no audio recordings were taken. This could have led to limitations in contextualising quotes, making to challenging to understand. However, simultaneous documentation was done by one of the researchers (SK) who was also working on the analyses. Moreover, with eleven workshops allowing for an elaborate circulatory process, a large number of sessions using pinboard cards, and posters resulted in a large collection of data providing extensive material for constant comparisons during analysis and to mirror findings with the participants [71].

In the quantitative aspect of this study, it was not possible to ascertain if the time spent engaging with material was efficiently used. Additionally, in the workshops, the chance to evaluate the training through reflection on the approach and learning value might have been missed. Hence this study could be replicated with a different approach. Perhaps with a control group in an experimental design, removing confounding variables which might affect the results due to the aforementioned point or with reflection sessions on the training for the qualitative aspect to maximise the learning and teaching content.

If these results are to be generalised to experienced physiotherapists in other contexts, caution should be exercised because this study was conducted in Germany among a homogenous group of bachelor physiotherapists with a limited sample size and response rate. However, keeping these things in mind, we think that the study’s rigor allows for some interesting comparisons to be drawn based on its findings.

## Conclusion

In this study, it was found that the quality of engagement in learning has a correlation with participants’ evaluation performance. Training strategy, interest in the subject and quality of learning materials could contribute to the long-term sustainability of learning outcomes.

The presented blended learning strategy on stratified care was valuable and interesting both on the side of the students and the course developers. The time spent developing the course and delivering it for two consecutive years produced measurable outcomes with good potential for translation into practice. The training achieved reasonable participation and stimulated participants’ interests. PreTest kinesiophobia and participants’ interest in the topic were two crucial factors that predicted outcomes after training. Participants who enjoyed the course and spent more time engaging the resources had better overall results. The findings from this study indicate that the blended learning strategy has good potential to positively influence physiotherapists’ attitudes and develop competency in practice. Participants resolved to further apply specific aspects relating to communication in their practice. This training further gives an impulse to enhance physiotherapeutic education, treatment and provides a template for training that can be evaluated in external cohorts.

## Electronic supplementary material

Below is the link to the electronic supplementary material.

Supplementary Material 1

Supplementary Material 2

Supplementary Material 3

Supplementary Material 4

Supplementary Material 5

Supplementary Material 6

Supplementary Material 7

## Data Availability

Qualitative data generated or analysed during this study are included in this published article [and its supplementary information files]. The quantitative datasets generated and/or analysed during the current study are not publicly available (because they contain identifiable information) but are available from the corresponding author when providing a scientifically sound study design.

## Abbreviations

SRT: Self-reflection test
SC: Stratified Care
LBP: Low back pain
Class2020: First cohort
Class2021: Second cohort
SBT: STarT-Back Tool
MSK: Musculoskeletal
EV: Evaluation question
RQDA: R-Qualitative Data Analysis
NSLBP: Non-specific low back pain
OpenOLAT: Open Online Learning and Training
TSK-PT: The Tampa Scale for Kinesiophobia for Physiotherapists
PreTSK-PT: Tampa scale for kinesiophobia completed by participants before intervention
PostTSK-PT: Tampa scale for kinesiophobia completed by participants after intervention
