# Supplementary material for: Evaluation of a blended learning approach on stratified care for physiotherapy bachelor students
Source: BMC Med Educ. 2023 Jul 31;23:545. doi: 10.1186/s12909-023-04517-5 (PMC10391990; doi:10.1186/s12909-023-04517-5)
Supplement: Supplementary file 1 — Supplementary Material 1 [file 12909_2023_4517_MOESM1_ESM.docx]

| **Stratified Care Training and Workshop** |
| --- |
| ***Learning outcomes: Participants will;***   - become familiar with the general overview, benefits and aims of SC - be conversant with the Theoretical background to SC - become aware of biopsychosocial approach - learn the development, prognosis and progress of NSLBP - learn about treatment using SC in patients with NSLBP - discuss and reflect on experiences and perception about using SC in patients with NSLBP |

# Additional file 1: Training objectives

SC; Stratified care, NSLBP; Non-specific low back pain
