# Supplementary material for: Evaluation of a blended learning approach on stratified care for physiotherapy bachelor students
Source: BMC Med Educ. 2023 Jul 31;23:545. doi: 10.1186/s12909-023-04517-5 (PMC10391990; doi:10.1186/s12909-023-04517-5)
Supplement: Supplementary file 2 — Supplementary Material 2 [file 12909_2023_4517_MOESM2_ESM.docx]

| **Key questions** | **Maintenance question** | **Concrete questions** |
| --- | --- | --- |
| 1. What **crosses your mind** when you think about stratified care and clinical practice? | - What barriers? - What enablers? - Is there anything else? | - What aspects of SC did you find easy to apply practically in the clinics? - What can you say about the strength of the evidence supporting SC - How do you think it can be adapted to suit local needs? - What can you say about the cost of implementation? - What is your opinion about having this competency for all physiotherapists in Germany? - What can you say about the ease of assessing information on SC? |
| 2.Talk about your **experiences** with Stratified care in clinical practice | - What barriers? - What enablers? - Is there anything else? | - What experiences show the effect of this knowledge on your practice - How do you think your patients benefited from your competence? - How did this knowledge of SC impact on interprofessional collaboration? - How did your practice organisation help or hinder the application of SC at work? - How did this knowledge affect your confidence in treating patients with LBP? - What impact does practice have on your understanding of stratified care |
| 3.What is your overall opinion about the **training** on stratified care? | - What barriers? - What enablers? - Is there anything else? | - How does this knowledge relate to previous knowledge and experiences? - How important is the knowledge of SC to you? - How will you recommend SC for a colleague? - What skills enable you to properly practice SC? |

# Additional file 2: Interview guideline

SC: Stratified care, LBP: Low back pain.
