# Supplementary material for: Evaluation of a blended learning approach on stratified care for physiotherapy bachelor students
Source: BMC Med Educ. 2023 Jul 31;23:545. doi: 10.1186/s12909-023-04517-5 (PMC10391990; doi:10.1186/s12909-023-04517-5)
Supplement: Supplementary file 3 — Supplementary Material 3 [file 12909_2023_4517_MOESM3_ESM.docx]

| **Set** | **N=62** | **Areas of enquiry** | **New codes** | **Category** |
| --- | --- | --- | --- | --- |
| 04_11_2020 | 5 | - - Working with colleagues/mentorship   - Working with a challenging boss   - Patient and PT relationship | - - Documentation of successes   - Feedback from patients | - - Facilitators   - Perception of training   - Strategies   - Challenges with tool |
| 01_11_2020 | 4 | - - Reasons for non-use of approach/tool   - Training of physiotherapists   - Further strategies for working with a challenging boss | - - Time constraints   - Training physiotherapists | - - Facilitators   - Perception of training   - Strategies   - Challenges with tool |
| 18_11_2020 | 5 | - - Dealing with issues with data privacy   - Documentation-Anonymity, licence | - - Self-reflection   - Planning   - Patient education | - - Facilitators   - Perception of training   - Strategies   - Challenges with tool |
| 25_11_2020 | 5 | - - Perception of training   - Interests and applicable areas   - Frustration with practice and outcome | - - Finance   - Questionnaire in routine use   - Pre-printed versions | - - Facilitators   - Perception of training   - Strategies   - Challenges with tool |
| 02_12_2020 | 5 | - - Use of e-formats of the SBT   - Covid situation | - - Digitalisation | - - Facilitators   - Perception of training   - Strategies   - Challenges with tool |
| 10_11_2021 | 6 | - - Ideas on financial aspects   - Ideas on use in other specialities besides MSK | - - Outside the scope of the approach | - - Perception of the approach |
| 17_11_2021 | 9 | - - What familiar aspects could one use?   - Aspects regarding communication with colleagues/boss | - - Passive-assistive support | - - Experiences |
| 01_12_2021 | 6 | - - Ideas on modernisation of training   - Ideas on how to inform colleagues   - Ideas on interprofessional groups | - - Use of standardised tools/guidelines   - Delegate tasks to professional groups | - - Strategies   - Strategies |
| 08_12_2021 | 6 | - - Ideas on communication with colleagues   - Ideas on change in physiotherapists | - - Change in physiotherapists   - Group therapy | - - Challenges with approach   - Strategies |
| 15_12_2022 | 7 | - - The physician in this model, where?   - Ideas on convincing colleagues and patients | - - Physicians as barriers | - - Challenges with approach |
| 05_01_2022 | 4 | - - Ideas on internal training/mentorship   - Ideas on relevance of experience or degree in practice?   - Ideas on dealing with challenges with doctors | - - Physicians as enablers   - Training should be broadened   - Focus on younger PTs   - Lack of regular questionnaire usage | - - Facilitators   - Perception of training   - Strategies   - Challenges with tool |

**Additional file 3: Qualitative findings from workshops**

PT: Physiotherapist, SBT: STarT-Back Tool, MSK: Musculoskeletal
