# Supplementary material for: Evaluation of a blended learning approach on stratified care for physiotherapy bachelor students
Source: BMC Med Educ. 2023 Jul 31;23:545. doi: 10.1186/s12909-023-04517-5 (PMC10391990; doi:10.1186/s12909-023-04517-5)
Supplement: Supplementary file 4 — Supplementary Material 4 [file 12909_2023_4517_MOESM4_ESM.docx]

**Additional file 4: T-test for significant difference in training statistics between groups**

|  | | | | **Total** | | |
| --- | --- | --- | --- | --- | --- | --- |
|  |  |  |  | Mean (SD) | 95% CI | |
|  |  |  |  |  | Lower | Upper |
| **Exam Score** | | | | 17.04(5.41) | 15.67 | 18.42 |
| **Time** | | | | 8078.86(6960.68) | 6539.73 | 9618.00 |
| **Score** | | | | 30.01(13.94) | 26.93 | 33.10 |
| **Attempts** | | | | 11.15(6.81) | 9.64 | 12.65 |
| **SRT Time** | | | | 3606.25(2139.10) | 3130.22 | 4082.28 |
|  | **SRT1 Time** | | | 2148.18(1649.10) | 1793.34 | 2501.89 |
|  | **SRT2 Time** | | | 1496.09(1054.05) | 1277.05 | 1758.89 |
| **SRT Score** | | | | 27.04(4.74) | 25.98 | 28.09 |
|  | | | **SRT1 Score** | 11.08(2.295) | 10.58 | 11.58 |
|  |  |  | **SRT2 Score** | 16.37(2.523) | 15.87 | 16.91 |
| **SRT Attempts** | | | | 2.66(1.16) | 2.40 | 2.92 |
|  | | **SRT1 Attempts** | | 1.40(0.902) | 1.23 | 1.64 |
|  |  | **SRT2 Attempts** | | 1.29(0.705) | 1.15 | 1.46 |

CI: 95% Confidence Interval for Mean

SRT; Self-reflection Test. class2020: First cohort. class2021: Second cohort
