# Supplementary material for: Evaluation of a blended learning approach on stratified care for physiotherapy bachelor students
Source: BMC Med Educ. 2023 Jul 31;23:545. doi: 10.1186/s12909-023-04517-5 (PMC10391990; doi:10.1186/s12909-023-04517-5)
Supplement: Supplementary file 5 — Supplementary Material 5 [file 12909_2023_4517_MOESM5_ESM.docx]

**Additional file 5: Participants Perception of Training on Stratified Care**


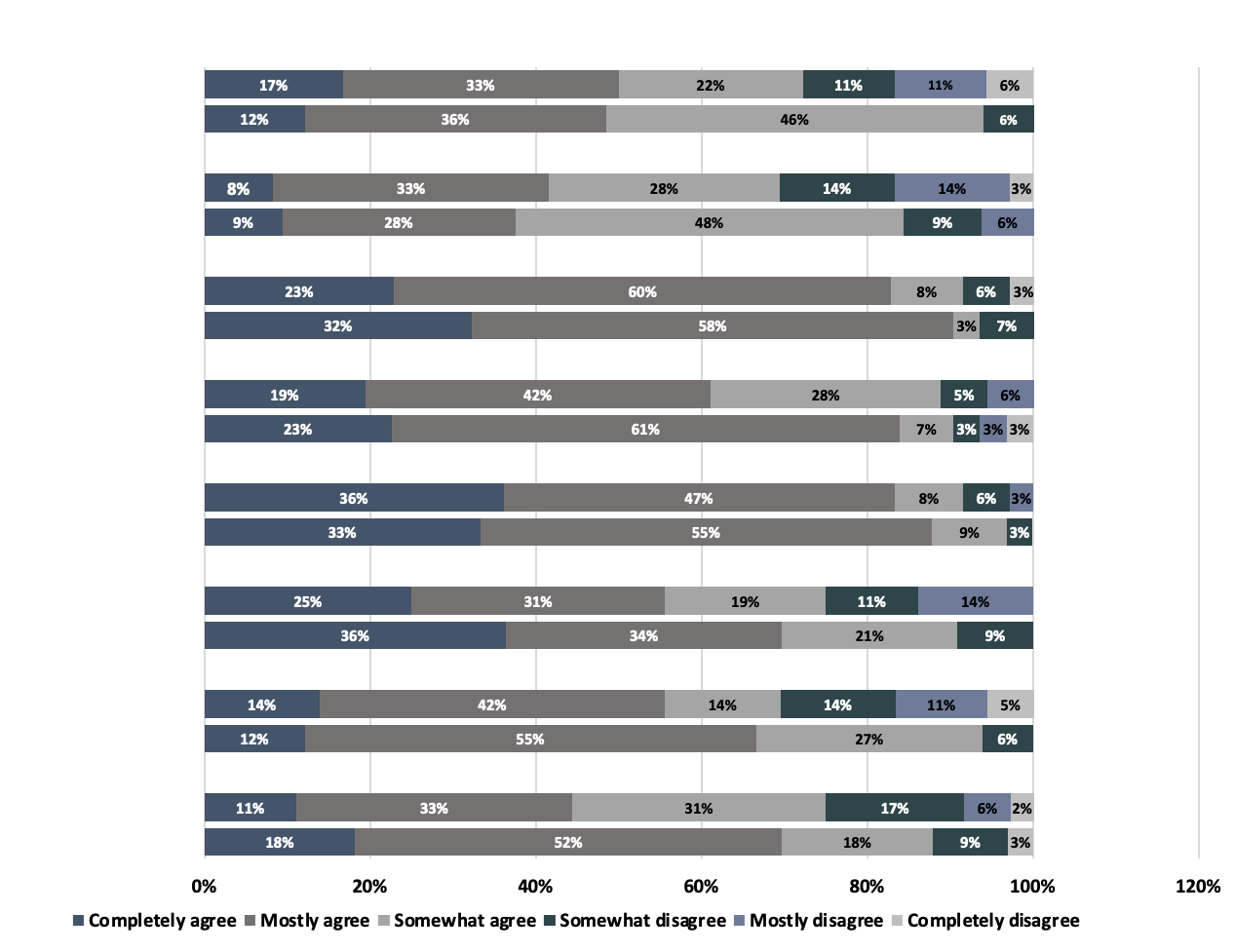


**The content in this**

**course was current**

**The relevance of the course**

**content offered was high**

**Additional helpful resources have been added**

**I rate my learning gain**

**from this course highly**

**The course was clearly structured**

**The learning objectives of the course were clearly defined**

**The course furthered my Interest in the subject**

**The material was appropriately illustrated**

**Class 2021**

**Class 2020**

**Class 2021**

**Class 2020**

**Class 2021**

**Class 2020**

**Class 2021**

**Class 2020**

**Class 2021**

**Class 2020**

**Class 2021**

**Class 2020**

**Class 2021**

**Class 2020**

**Class 2021**

**Class 2020**

**LEGEND: Class2020; First cohort, Class2021; Second cohort**
