# Supplementary figures and images for: Evaluation of a blended learning approach on stratified care for physiotherapy bachelor students
Source: BMC Med Educ. 2023 Jul 31;23:545. doi: 10.1186/s12909-023-04517-5 (PMC10391990; doi:10.1186/s12909-023-04517-5)

**Additional file 6: Inductively developed strategies for implementation from Workshops
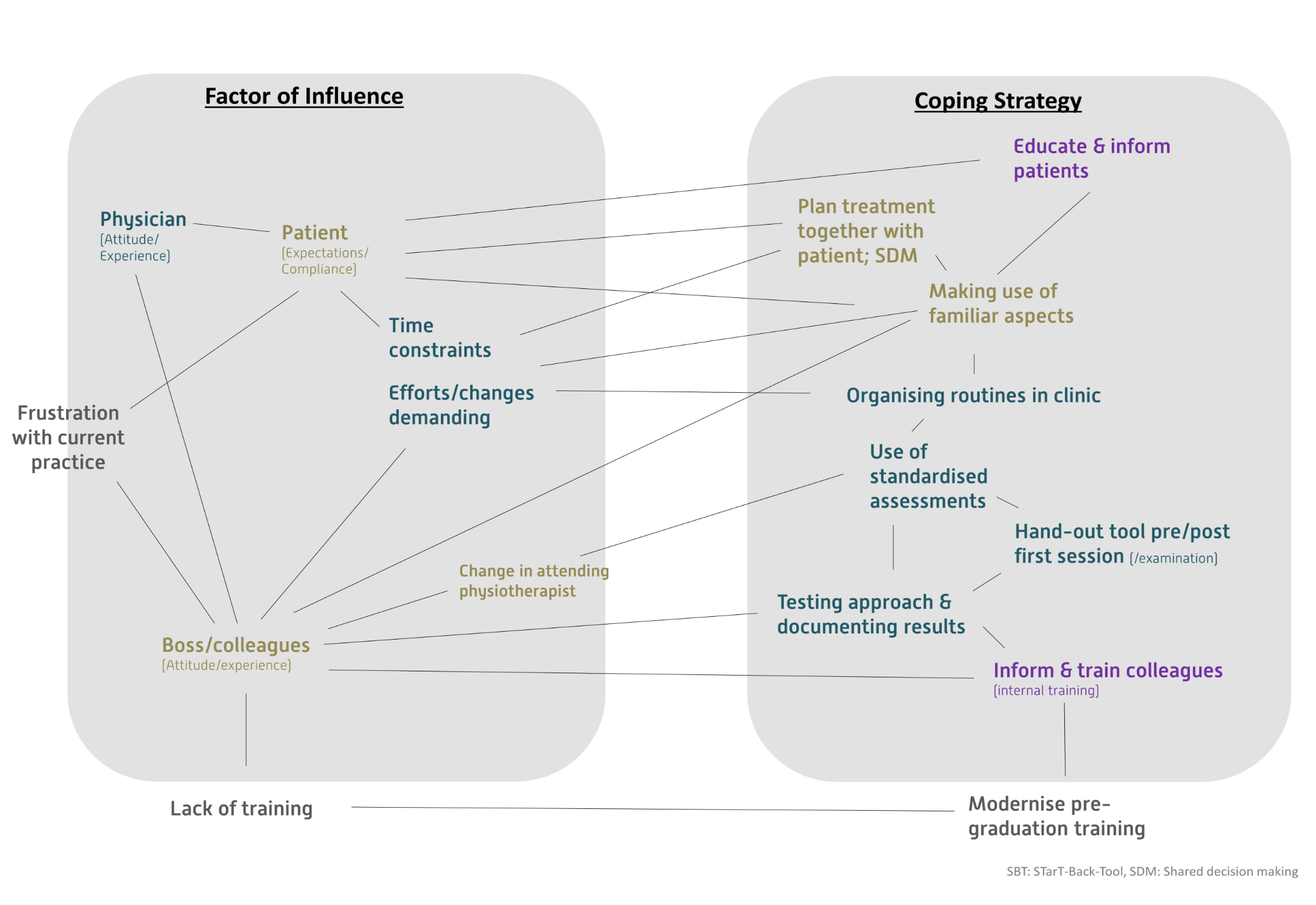
**

Supplement: Supplementary file 6 — Supplementary Material 6 [file 12909_2023_4517_MOESM6_ESM.docx]
