# Supplementary material for: Evaluation of a blended learning approach on stratified care for physiotherapy bachelor students
Source: BMC Med Educ. 2023 Jul 31;23:545. doi: 10.1186/s12909-023-04517-5 (PMC10391990; doi:10.1186/s12909-023-04517-5)
Supplement: Supplementary file 7 — Supplementary Material 7 [file 12909_2023_4517_MOESM7_ESM.docx]

**Additional file 7: Inductively developed categories, sub-categories, coded and quotes from Workshops**

| **Categories** | **Sub-categories** | **Codes** | **Quotes** |
| --- | --- | --- | --- |
| Evolving to maturity in practice | Self-advancement | Used aspects of the approach | G1P4: I have not directly used this approach actually in clinical practice. Used indirectly in other ways |
|  |  | Learned to change focus of treatment | G2P2: I have learned to focus on getting Patients active and paying attention to their complaints more. |
|  |  | Learned to listen differently | G5P3: Noting when patient gave comments. My patient method communication |
|  |  | Experienced psychological factors in patients | G4P1: I have seen the influence of psychosocial factors in clinical practice, mainly catastrophising |
|  |  | Positive result from using SC in practice | G3P4: To some extent. I have much LBP patient and I have helped them get active and encouraged them. |
|  | Individual demands | Did not use approach due to work stress | G3P2: I could not use the approach due to work stress. where I work mainly blue-collar workers visit and I can't spend much time in treatment. |
|  |  | Corona situation affects use | G8P4: The corona situation makes it more difficult in the clinics. |
|  |  | Not many LBP patients | G4P1: Hardly any lumbar spine problems in the patients I handled |
|  |  | Getting used to the approach | G5P4: I need [opportunities] to get used to the approach. |
| Perceiving the determinants of stratified care | Factors affecting use of the approach | Physicians as enablers | G10P2: Describing the characteristics of the doctor-is he knowledgeable or not. Knowledge education and exposure  G4P3: Physicians inhouse helps to communicate easier when they know about the tool. |
|  |  | Physicians as barriers | G10P3: Barrier, when they get referral as form of communication. the precision of information unhelpful |
|  |  | Change in PT | G3P5: In the clinics therapists change [new therapist for an old patient] every day, can influence outcome and use. |
|  |  | Time as barrier | G2P4: Time constraints, longer treatment time usually not available. |
|  |  | Time as enabler | G3P2: Positive aspects of the approach are that it saves time and is efficient |
|  |  | Training | G1P5: Specialised pain therapy is vital for high risk patients. |
|  |  | Money as barrier | G1P2: 45 mins treatment is difficult to implement with health insurance policy  G6P3: PTs having an Economic view of practice |
|  |  | Money as enabler | G3P5: Positive aspects was that the approach is […] cost-effective. |
|  |  | Colleagues as barriers | G2P3: Lack of support from colleagues |
|  |  | Colleagues as enablers | G3P4: Support from colleagues are enablers. I find the relationship with EBP interesting and I'm sure other colleagues will appreciate another perspective |
|  |  | Setting | G11P3: SBT has little relevance on Neurologic and Paediatric Patients. |
|  |  | Patient attitude/Expectations as barrier | G4P3: Working with long term patients. Patients expect to get massage. |
|  |  | Patient attitude/Expectations as enablers | G5P5: In guiding Patients to be more active some patient might be compliant and this is an enabler. Co-operative Patients. |
|  | Factors affecting use of the SBT | Lack of regular questionnaire usage | G8P3: Little use of questionnaires [in general] by therapists |
|  |  | Questionnaire makes PT job easier | G2P2: The questions are understandably formulated  G2P3: Good and well-organised tool, it is well ordered and arranged  G4P1: The SBT makes PT job easier |
|  |  | Other tools | G3P3: Negative aspects are that there are already multiple [assessment] tools/instruments to choose from. |
|  |  | More effort needed | G1P4: On the other hand, it is additional work for clinicians and PT |
| Strategising for implementation | Adaptive strategies | Using familiar aspects | G1P1: Adapt Anamnesis |
|  |  | Organise routines in the clinic | G6P2: Integration of questionnaires in clinical routine is an enabler. |
|  |  | Test and document results | G4P3: Documenting outcome after using it in clinical practice. |
|  |  | Issue SBT before the first treatment and include in intake. | G3P4: Handing over the questionnaire before treatment, many are okay with this extra questionnaire while others are unnerved by it. Some tick the questionnaire willingly but quickly just to get over with it while some are more detailed in filling it.  G2P4: fill out the questionnaire on or before first contact  G5P4: Digitalised assessment. Online tools for assessment should be used |
|  |  | Use of standardised assessments | G3P6: Standardised instruments can be used to overcome changes in [treating] therapists |
|  |  | Create treatment plans with patients | G5P5: Define clearly the aims and objective of treatment to Patients. Creation of a plan together with the Patient. |
|  | Targeting stakeholders | Educate and inform patients | G4P1: A lot of explaining as understandable as possible for the Patient. |
|  |  | Reach a consensus with patients | G2P4: Compromising approach to the activity if the candidate refuses at first |
|  |  | Interprofessional collaboration | G4P5: Interprofessional implementation G1P5: Specialised pain therapy is vital for high risk patients. Interprofessional team  G1P1: Interprofessional collaboration  G3P6: Difficult interprofessional perspective. |
|  |  | Modernise training | G1P2: Training on the use of the questionnaire. |
|  |  | Inform and train colleagues | G2P5: Uniform approach of the therapists by internal training |
| Adopting an outlook for future practice | Impression on Training | Easy to apply | G2P4: Not too much more complicated workload. Easy to use |
|  |  | Enhances practice | G5P3: Helps Organisation in practice.  G5P3: Content from basic training was strengthened. Responsive and interesting |
|  |  | Boosts confidence of new clinicians | G5P4: Directs young PT. It boosts the confidence of the therapist |
|  |  | Self-learning was useful | G5P2: Positive aspects are that we worked on a lot of topics by ourselves |
|  |  | It was lacking in concrete structure | G5P1: Perception of Training. Needs more visible structure. |
|  |  | Training should be broadened | G1P1: Would be nice to integrate this in other training.  G6P1: I feel you should have a bigger forum to discuss involving other specialities. Involve higher authorities, other students. |
|  | Resolve for application | Communication | G5P1: Communication from Patients, contextual relevance, communication with other patients was an interesting aspect.  G10P3: Patient need to be able to express themselves freely without being judged |
|  |  | Treatment planning | G2P1: A positive factor is planning therapy, being prepared to use the tool and hand it to the patient gives you a better idea of how to plan patient treatment. |
|  |  | Fastrack decision making | G3P4: Fast conclusion, finding solutions fast. Stratification. Fast track clinical decision making. |

PT: Physiotherapist, SBT: STarT-Back Tool, MSK: Musculoskeletal, LBP: Low Back Pain
